# Validation of a software application using electronic health records for automatic detection of community onset sepsis

**DOI:** 10.1038/s41598-025-99879-9

**Published:** 2025-05-12

**Authors:** Cristian Duré, Sandra Jonmarker, Eva Joelsson-Alm, Hampus Nordqvist, Katarina Bohm, Liivi Rimling, Mikael Andersson Franko, Maria Cronhjort, Kristian Ängeby

**Affiliations:** 1 Department of Clinical Science and Education, Karolinska Institutet, Stockholm, Sweden; 2 Department of Emergency Medicine, Capio S:t Görans Hospital, Stockholm, Sweden; 3 Department of Anaesthesia and Intensive Care, Södersjukhuset, Stockholm, Sweden; 4 Department of Infectious Diseases, Södersjukhuset, Stockholm, Sweden; 5 Department of Emergency Medicine, Södersjukhuset, Stockholm, Sweden; 6 Department of Clinical Sciences, Danderyd Hospital, Karolinska Institutet, Stockholm, Sweden

**Keywords:** Sepsis, SOFA Sequential Organ Failure Assessment, EHR Electronic Health Record, EDs Emergency Departments, ICUs Intensive care units, Bacterial infection, Medical research, Signs and symptoms

## Abstract

Our aim was to design and validate a software application, based on the Sepsis-3 criteria, capable of retrospectively identifying community-onset sepsis among emergency department patients requiring hospital admission.The application was developed using QlikView (Qlik, King of Prussia, PA, USA) software, and accessed data from the electronic health records TakeCare (CompuGroup Medical, Koblenz, Germany), and CliniSoft (CliniSoft, Kuopio, Finland). The application utilized indicators such as blood culture data, antibiotic administration, and Sequential Organ Failure Assessment scores to detect sepsis cases according to Sepsis-3 criteria. The application was tested retrospectively against a cohort from a large city hospital in Stockholm over a 2-year period, and its performance was compared to physician record reviews in a subset of cases identified by stratified random sampling. The results showed that among 229,195 emergency department visits leading to 60,213 hospital admissions, the application detected 7027 cases of sepsis. Validation using physician record review of a random selection of 426 cases demonstrated a sensitivity, specificity, positive predictive value, and negative predictive value of 95%, 99%, 92%, and 99%, respectively. The lower respiratory tract was the most common site of infection. This software application effectively identified community-onset sepsis patients using electronic health record data with high performance. It has the potential to improve sepsis identification as it operates independently of diagnostic codes and may, therefore, facilitate research in many areas of sepsis. Furthermore, it can be used as a tool within the healthcare system to enhance sepsis surveillance and evaluate quality improvement interventions.

## Introduction

The World Health Organization recognizes sepsis as a significant global health issue, being a leading cause of morbidity and mortality worldwide^1^. The definition of Sepsis has evolved over time and is now, according to the Third International Consensus Definition for Sepsis and Septic Shock, considered to be a life-threatening organ dysfunction caused by a dysregulated host response to infection^2^. Organ dysfunction is identified through the scoring system, Sequential Organ Failure Assessment (SOFA)^3,4^.

The real burden of sepsis is difficult to estimate. Although the International Classification of Disease (ICD) coding method is the most practical tool to measure sepsis incidence at scale, this method has been proven unreliable when compared to medical record review. A recent study found that the incidence of sepsis among hospitalized patients, based on the Sepsis-3 criteria, was 4.1%, as determined by medical record reviews, while the incidence based on ICD-10 codes was only 1.0%^5^. Intensive care Unit (ICU) patients had higher rates of correct coding, maybe because ICU doctors are more commonly caring for septic patients and thus more aware of the diagnosis. Other factors affecting the accuracy of ICD coding for sepsis are severity of disease, community-aquired or nosocomial sepsis, time and source of sepsis. A study from Hong Kong showed that using administrative data tends to underestimate the local burden of sepsis when compared to Electronic Health Record (EHR)-based methods, probably due to inadequate training and resources to perform the coding^6^.

The rational for developing this application was to accurately identify true sepsis patients, enabling epidemiological studies, monitoring of incidence to improve planning of health care, and evaluation of quality improvement interventions.

We have developed a new software application to identify patients with sepsis in the EHR system TakeCare (TC) (CompuGroup Medical, Koblenz, Germany), generally used for hospital and outpatient records, as well as Clinisoft (CCC) (CliniSoft, Kuopio, Finland), a patient data management system (PDMS) used in Intensive Care Units (ICUs).

This software application retrieves data from a large patient cohort. Since this process can be automated, it has the potential to be nearly as efficient as using ICD codes, but potentially more reliable.

The objective of this study was to evaluate the diagnostic accuracy of this fully automated software application to identify patients with community-acquired sepsis using physician medical record review as the reference standard.

## Results

Out of the 229,195 patient visits to the emergency department (ED), 60,213 resulted in hospitalization. The application identified 7027, visits (3.1%) as cases of sepsis. Out of these, 3099 (44%) were female and the median age was 74 years. The maximum SOFA-score achieved was a median of 3 points. A total of 732 (10%) of the sepsis visits resulted in an admission to the ICU. Additional descriptive data are outlined in Table 1. Except for bilirubin, measuring the liver function component of the SOFA score, there was minimal missing data in the SOFA scores (Table 2).

**Table 1:** Additional descriptive data for patients in Group A (meeting the Sepsis 3-criteria by the application), Group B (Suspicion of a serious infection but not Sepsis by the application) and Group C (Low likelihood of sepsis or serious infection by the application).

	Group A n: 7 027	Group B n: 1 958	Group C n: 51 228
Age in median (IQR), years	71 (84–63)	56 (72–39)	69 (80–54)
Female sex, n (%)	3 099 (44)	1 041 (53)	25 630 (50)
ICU admissions, n (%)	732 (10)	18 (0.9)	N/A
90-day mortality, n (%)	1 335 (19)	58 (3.0)	N/A
SOFA score in median	3	1	N/A

*N/A* non applicable.

**Table 2:** Extent of missing data in the SOFA score, broken down by each component in Group A and Group B, as indicated by the application.

	Group A n: 7027 (100%)	Group B n: 1958 (100%)
Cardiovascular	n: 7 (0.1%)	n: 1 (0.05%)
Neurologic	n: 29 (0.4%)	n: 1954 (0.2%)
Coagulation	n: 10 (0.1%)	n: 5 (0.3%)
Liver	n: 3469 (49.4%)	n: 1310 (66.9%)
Renal	n: 50 (0.7%)	n: 148 (7.6%)
Respiration	n: 4 (0.06%)	n: 25 (1.3%)

According to the reviewers’ medical record review, a total of 129 visits of 140 in group A fulfilled the true Sepsis-3 criteria while 11 were deemed false positive. Four visits of 143 in group B and 1 visit of 143 in group C fulfilled the true Sepsis-3 criteria and these were thus considered to be false negative. Based on these figures, the QV application achieved a sensitivity of 0.95 (95% CI 0.76–0.98), a specificity of 0.99 (95% CI 0.98–0.99), positive predictive value 0.92 (95% CI 0.86–0.96) and negative predictive value 0.99 (95% CI 0.96–1.00). The positive likelihood ratio was 91 (95% CI 56–199), the negative likelihood ratio was 0.061 (95% CI 0.002–0.16) and Youden’s index was 0.93 with (95% CI 0.83–0.99) (Table 3 and Supplemental File 1).

**Table 3:** Statistical performance of the evaluated QlikView application for detecting sepsis compared to physician record review as reference standard.

Sensitivity (95% CI)	0.95 (0.76–0.98)
Specificity (95% CI)	0.99 (0.98–0.99)
Positive predictive value (95% CI)	0.92 (0.86–0.96)
Negative predictive value (95% CI)	0.99 (0.96–1.00)
Positive likelihood ratio (95% CI)	91 (56–199)
Negative likelihood ratio (95% CI)	0.061 (0.002–0.16)
Youden’s index (95% CI)	0.93 (0.83–0.99)

*CI* Confidence Interval.

The false positive patients in group A, were further reviewed. One patient had a SOFA score of 2 points due to trauma, while another experienced respiratory failure unrelated to infection. Four patients initially had a baseline SOFA score of 1 point, which increased to 2 points at time zero, and therefore did not meet the criterium of an increase of two SOFA scores. Additionally, one patient had myelodysplastic syndrome, leading to a high SOFA score. Another patient failed to meet the infection criteria, and one received an elevated SOFA score due to an error in GCS (Glasgow Coma Scale) recording. Furthermore, one patient had a high SOFA score due to dehydration, and the last patient experienced a stroke followed by seizures, resulting in increased SOFA score. Initially, in all cases, sepsis could not be ruled out, so antibiotics were started as a precaution but were discontinued shortly after admission.

In group B, the four false negative patients had a SOFA score attributed to respiration, which the application failed to recognize due to inaccuracies in the patient records regarding the amount of oxygen administered. This information was not entered correctly into the EHR. Regarding the false negative patients in group C, one individual had been blood-cultured in a geriatric ward prior to transfer to the emergency department, where new blood cultures were not obtained (Table 4).

**Table 4:** Cases of sepsis detected with the software application compared to health record review.

		Group A (n = 140) Cases considered to have sepsis according to the software application (infection likely, SOFA score ≥ 2)	Group B (n = 143) Cases *not* considered to have sepsis according to the software application (infection likely, SOFA score < 2)	Group C (n = 143) Cases *not* considered to have sepsis according to the software application (serious infection or sepsis not likely)
Sepsis according to health record review (i.e. “true sepsis”)	Yes	129 (true positive)	4 (false positive)	1 (false positive)
	No	11 (false positive)	139 (true negative)	142 (true negative)

Infection characteristics of the patients in group A (n = 129) can be seen in Table 5. The most common site of infection was lower respiratory tract (41/129) followed by urinary tract (31/129). Among subjects in group A that fulfilled the true sepsis criteria, 60% fulfilled the criteria for possible infection, 22% for probable and 19% for definite infection and 16% were classified as possible infection due to unknown or uncertain source of infection (Table 5).

**Table 5:** Infection characteristics of 129 patient visits considered to have sepsis both by the QlikView application and by medical record review.

Source of infection	Likelihood of infection			
	Definite, n	Probable, n	Possible, n	Total, n (%)
Lower respiratory tract	8	11	22	41 (31.5%)
Urinary tract	7	12	12	31 (24.0%)
Gastrointestinal tract	3	2	14	19 (14.5%)
Skin and soft tissue	1	3	7	11 (8.5%)
Bones and joints	1	0	1	2 (2.5%)
Cardiovacular	1	0	0	1 (0.7%)
Intravenous catherter related	1	0	0	1 (0.7%)
Dental infection	1	0	0	1 (0.7%)
Psoas abscess	1	0	0	1 (0.7%)
Unknown	0	0	21	21 (16.2%)
Total	24 (18.6%)	28 (21.7%)	77 (59.7%)	129 (100%)

## Discussion

The application was designed to analyze a large clinical data set and provide a more reliable and consistent way of identifying septic patients from the EHR. We found that the application was able to capture 95% of sepsis visits, including those admitted to the ICU, with a specificity of 99%. Compared to a previous Swedish study, the application performs slightly better, and one possible reason could be the use of ICU data in addition to the data from EHRs^7^. Moreover, we found that 16.2% of admissions were classified as possible infection due to an unknown source of infection, highlighting that the application can detect cases of sepsis where diagnosis is difficult. Overall, the application has the potential to be used in all hospitals working with the EHR system TC and the PDMS system CCC, to facilitate further studies on sepsis incidence, mortality, and adherence to the treatment bundles of the Surviving Sepsis Campaign^8^.

Currently, the strategies used to register sepsis using ICD codes are not sufficient in accurately determining the true incidence of the condition^9^. In Hong Kong, EHR-based surveillance revealed a rise in standardized sepsis incidence and mortality at the population level from 2009 to 2018. This method proved to be significantly more accurate than administrative definitions^6^. These results highlight the feasibility and benefits of using an EHR-based approach for large-scale sepsis monitoring. Estimates derived from claims data may not accurately reflect the true clinical picture and can be influenced by variations in how diagnoses and codes are assigned over time^10^. For example, according to Rhee et al.^11^, if a hospital uses administrative codes to track sepsis, there is a high probability that they will see higher sepsis case counts and lower sepsis mortality rates. On the other hand, Swedish studies have shown that clinicians rarely use ICD-10 codes indicating sepsis, meaning that many cases would be missed and sepsis incidence probably underestimated^5^. Apparently, ICD-10 coding seem to differ substantially between different settings^12^. In the application developed for the present study, clinical data relevant for the Sepsis-3 definition has been used, ensuring a more correct detection of sepsis visits.

Using the application can help hospitals to measure the impact of their sepsis prevention and treatment initiatives and improve the quality of care for patients. This creates the potential for valuable feedback that can guide quality improvement interventions, such as educational programs, systems for earlier sepsis recognition, treatment bundles, and infection control measures.

One limitation of the study is that the application relies on data input by nurses and physicians, including clinical judgments such as decisions regarding blood cultures, antibiotic prescriptions, or initiation of vasopressor therapy. This reliance on individual practitioner judgments introduces a potential source of variability in the data. Additionally, there is no guarantee that all patients with sepsis underwent blood culture testing, meaning some cases may have been missed by the application.

Furthermore, there was a significant level of missing data regarding the liver function (bilirubin). Even though it is probable that physicians have been more likely to have ordered bilirubin in sicker patients or with a suspicion of liver engagement, it is still possible that a subset of these patients would have a SOFA score of ≥ 2 if bilirubin had been analyzed. Thus, these patients should have classified as group A and ultimately this could have resulted in an underestimation of the number of visits diagnosed with sepsis by the application This highlights the importance of adequately training physicians in conducting all tests contributing to the SOFA score in order to recognize patients with potential sepsis.

In relation to the timeframe for data collection, during the assessment of clinical criteria for the sepsis-3 definition, it was allowed for cultures to be drawn up to 24 h after the initiation of antimicrobial therapy, or for antimicrobials to be commenced up to 72 h after culture collection^13^. The initial intention of this study was to encompass all three time frames. However, during the early stages of application development, it became apparent that utilizing all three windows was intricate and time-intensive, particularly when cross-referencing with the EHR. There was a concern that such complexity might introduce errors into the validation process. Consequently, a decision was made to simplify the approach. We opted for a 48-h time window from the patient’s arrival at the ED, as utilized for the SOFA score, for the timing of both blood cultures and the administration of antimicrobials following the definition of community acquired sepsis^14^. However, given the requirement for at least two doses of antibiotics for sepsis treatment, only the initial dose needed to be administered within 48 h.

Another limitation is that, although the reviewing physicians independently assessed the medical records to determine sepsis or infection for all cases, they were not blinded to the application software. This could potentially introduce bias and impact the consistency of the results. However, we believe this effect is minimal, as a strict protocol was followed. Also, different reviewers took responsibility for the different validation groups. The participation of one researcher (CD) in the validation process across all three groups (A, B, and C), and the strict protocol should vouch for consistency of our results.

Also, the study was only conducted in one center. Even though the application could be used also in other hospitals utilizing TC and CCC systems, local validation will be needed to use the application in other hospitals..

The present study is limited in its scope to identify community onset sepsis, however, the application could be adapted to detect also hospital onset sepsis cases^15^.

## Methods

### Setting and study population

This study was conducted at Södersjukhuset (Stockholm South General Hospital), Sweden, which has one of the largest EDs in the Nordic countries. The study covers a period from May 1st, 2016, to April 30th, 2018. During these two years 229,195 adult visits were registered to the ED and out of these, 60,213 were admitted to the hospital.

### Electronic health records

We used the EHR system TakeCare (TC) (used for hospital and outpatient records) as well as Clinisoft (CCC), a patient data management system used in ICUs. Both systems store and retrieve clinical information, including patient history, vital signs, laboratory results, drug administration, other treatments provided, and diagnosis codes.

### Application development

A software engineer developed the application in the QlickView (QV) software (Qlik Technologies Inc, King of Prussia, PA, USA.), enabling QV to retrieve and store data from TC, as well as from CCC. Data was generated, coded, and analyzed from a population that consisted of all adult patient visits (≥ 18 years of age) to the ED. The patients were monitored for the initial 48-h period or until they were discharged or passed away, whichever occurred first.

To identify patients likely to have community-onset sepsis according to the Sepsis-3 criteria, the QV application was programmed to identify patients who (1) had blood cultures taken within 48 h from the arrival to the ED, (2) received two doses of antibiotics used for sepsis, and (3) obtained a SOFA score ≥ 2 during at least one 24 h window within 48 h from arrival to the ED. “Time zero”, i.e. the exact time when a patient was considered to have sepsis was defined as the first time a patient obtained a SOFA score ≥ 2. Antibiotics used for sepsis were defined by the recommendations of the Swedish Society of Infectious Diseases (SILF) for treating community onset sepsis in Sweden, namely benzylpenicillin, cloxacillin, piperacillin + tazobactam, cefotaxime, ceftazidime, meropenem, imipenem + cilastatin, tobramycin, gentamicin and amikacin^16^.

Two doses of antibiotics used for sepsis were required; the first should be administered within 48 h from the patients’ registration at the ED, aiming to identify community-acquired sepsis^14^. The second dose had to be administered within 18 h from the first dose. The time frame was selected to ensure the inclusion of patients on a twice-daily regimen, even if there were delays in administering the second dose, as all antibiotics recommended by SILF for sepsis treatment require at least two doses per day. Also, the time between prescription and administration may be delayed due to the nurses’ workload.

Patients reaching a SOFA score ≥ 2 for the first time later than 48 h from admission were not included since these were considered as having a hospital acquired sepsis^15^. The SOFA score’s respiratory component was calculated using the Ellis equation^17^, converting the peripheral capillary oxygen saturation (SpO2) to partial pressure of oxygen (PaO2) when PaO2 from arterial blood was not available. GCS was not used as a SOFA score criterion for intubated (and thus sedated) patients admitted to the ICU. Urine output was not used as a criterion due to a lack of data. If a SOFA score component was lacking, the value was considered to be zero.

The application did not incorporate the calculation of the patient’s baseline SOFA score. This decision was made due to the potential risk of missing information from previous hospital contacts and the possibility of patients being admitted to other hospitals that do not utilize TC or CCC as their EHR systems.

### Validation process

**Fig. 1:**
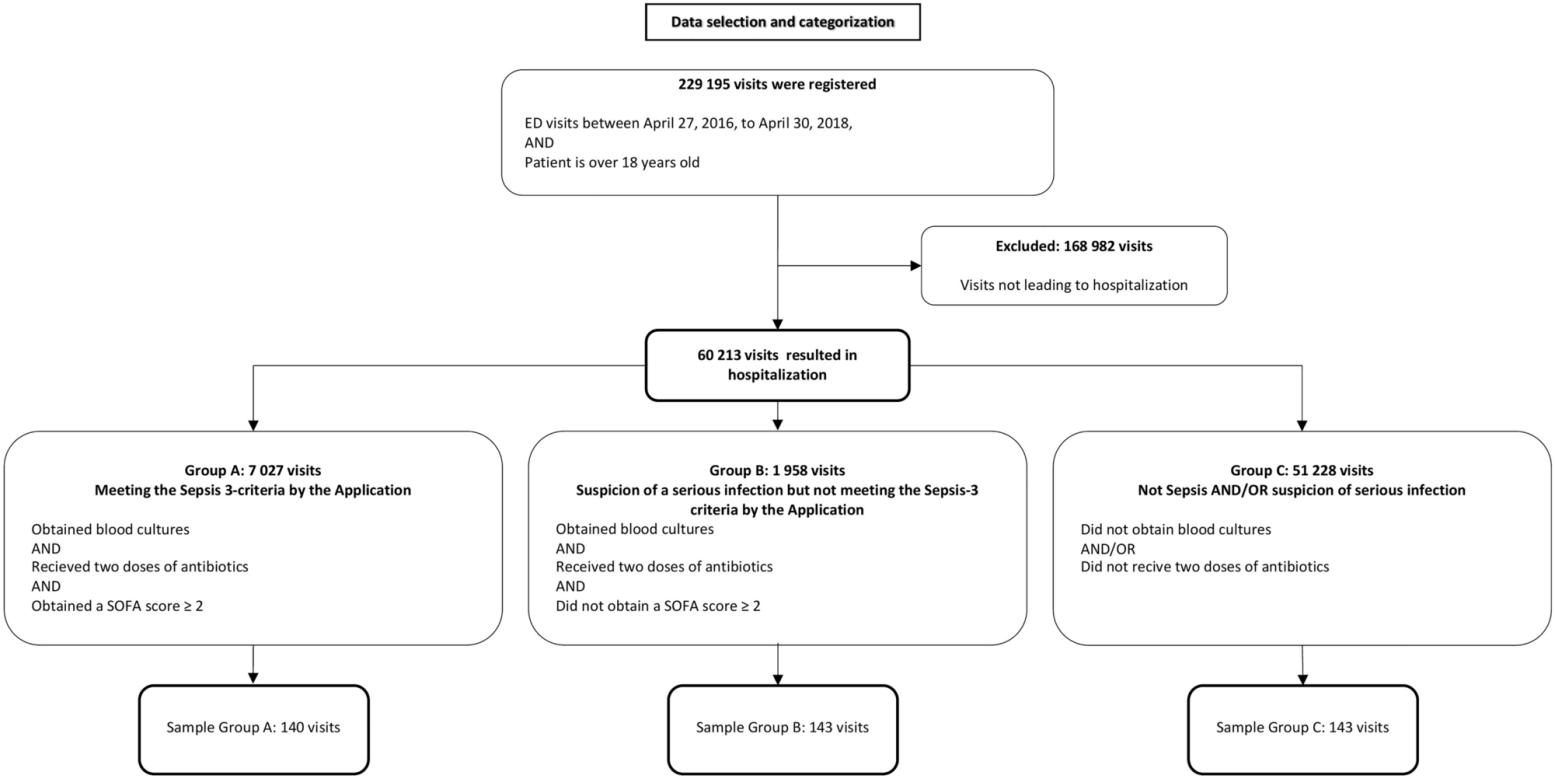
Data selection and categorization for Groups A, B, and C.

The QV software application was used for the entire cohort of the 60,213 consecutive visits to the emergency department that led to hospitalization. For validation of the application with medical record review, a stratified random sampling was made from three groups^18^. Stratification was made to ensure a sufficient number of patients from the relatively small groups A and B. From group A, consisting of 7027 ED visits considered to have sepsis according to the application, (i.e. blood cultures taken, antibiotics administered and a SOFA score of ≥ 2) a random sample of 140 visits was selected. From group B, consisting of 1 958 visits that had a suspicion of a serious infection but not sepsis according to the application (i.e. blood cultures taken, antibiotics administered but a SOFA score of < 2) a random sample of 143 visits was selected. Finally, from group C, consisting of 51 228 visits with low likelihood of sepsis or serious infection (i.e. not fulfilling the criteria for groups A and B) a random sample of 143 visits was selected. Thus, a total of 426 ED visits were selected by stratified random sampling from the total cohort and included for medical record review (Fig. 1).

Three emergency physicians, one licensed physician from the UK, and one ICU physician were tasked with reviewing whether patients met the Sepsis-3 clinical criteria. They conducted this assessment independently.

The application’s accuracy in detecting community-onset sepsis was compared to manual examination of the EHR.

The reviewers manually calculated baseline SOFA scores. For each component of the SOFA score, the baseline was determined as the best value measured within 90 days prior to arrival at the ED. If the value was unknown, it was assumed to be zero. The extent of missing data for the various SOFA components is detailed in Table 2. The manual examination revealed only one discrepancy between the missing data from the script and the missing data in the manual examination. In group A, the application identified five patients as having fewer points in the respiration SOFA compared to the manual examination, and the same occurred with one patient in group B. This discrepancy arose because the implementation of oxygen in some patients was only recorded manually by physicians or nurses, which the application could not detect in free text. However, in the manual examination, it was observed that all the six patients had a higher SOFA point when the manually recorded implementation of oxygen was added. The rest of the missing data did not present any discrepancy between the reviewers and the application.

Admissions that met the Sepsis-3 clinical criteria of having an increase in SOFA score of ≥ 2, as determined by physician review, were further evaluated for the likelihood of infection. For this purpose, the reviewers scrutinized clinical, microbiological and radiological findings of each patient. The episodes were classified into four categories: no infection, possible infection, probable infection, and definite infection, in accordance with the definitions outlined by Klouwenberg et al.^19^. Additionally, infections from an unknown source were included based on criteria established by Valik et al., defined as symptoms indicating an infection according to the attending physician, and the patient receiving a full course of anti-infective treatment, but no source could be determined. This category could only be classified as a possible infection. To be considered a true sepsis case in the assessment of the application’s sensitivity and specificity, patients had to meet Sepsis-3 clinical criteria (increase in SOFA score and the presence of possible, probable, or definite infection)^7^.

### Statistical analyses

Weighted estimates of sensitivity, specificity, Positive Predicted Value, Negative Predictive Value, and Youden’s index were used to account for bias caused by the stratified sampling scheme.

Confidence intervals with a 95% confidence level were calculated from 10 000 bootstrap samples^18^. Furthermore, the positive and negative likelihood ratios were calculated. Data handling and statistical analyses were conducted using R 4.1.1.

### Large language models (LLM), ChatGPT

A large language model, ChatGPT, was used solely for the purpose of correcting the English language in some parts of this manuscript; no content or images were created by the LLM.

## Conclusion

Our study demonstrates the feasibility of utilizing a software application, based on the Sepsis-3 criteria, to automatically identify patients with community onset sepsis, achieving high sensitivity, specificity, and predictive values in patients admitted through the emergency department. This application has the potential to improve sepsis identification as it operates independently of diagnostic codes and may, therefore, facilitate research in many areas of sepsis. Furthermore, it can be used as a tool within the healthcare system to enhance sepsis surveillance and evaluate quality improvement interventions.

## Supplementary Information

Supplementary Information.

## Data Availability

The data used for this study is available upon request to Dr. Cristian Duré at cristian.dure@ki.se.
