# Supplementary material for: Validation of a software application using electronic health records for automatic detection of community onset sepsis
Source: Sci Rep. 2025 May 12;15:16412. doi: 10.1038/s41598-025-99879-9 (PMC12069618; doi:10.1038/s41598-025-99879-9)
Supplement: Supplementary file 1 — Supplementary Information. [file 41598_2025_99879_MOESM1_ESM.pdf]

Estimation of diagnostic accuracy with stratified sampling

The population is stratified in three groups: group A consists of 7,027 patients, group B of 1,958 patients and group C of 51,228 patients. Stratified random sampling revealed that 129 of 140 sampled patients from group A, 4 of 143 of sampled patients from group B and 1 of 143 sampled patients from group C had sepsis. An estimate of the total number of patients with sepsis in the population is obtained as the weighted sum

$$N_{\text{Sepsis}} = 129 \cdot \frac{7027}{140} + 4 \cdot \frac{1958}{143} + 1 \cdot \frac{51228}{143} = 6850$$

The application identified 7,027 patients in group A and an estimate of how many of these truly had sepsis is obtained

$$N_{\text{Application}} = 129 \cdot \frac{7027}{140} = 6475$$

Hence, sensitivity is estimated as

$$\text{Sens} = \frac{N_{\text{Application}}}{N_{\text{Sepsis}}} = \frac{6475}{6850} = 0.945$$

Specificity is estimated similarly, except that we have to consider patients who did not have sepsis i each group, respectively.

$$N_{\text{No sepsis}} = 11 \cdot \frac{7027}{140} + 139 \cdot \frac{1958}{143} + 142 \cdot \frac{51228}{143} = 53324$$

and

$$N_{\text{Application}} = 139 \cdot \frac{1958}{143} + 142 \cdot \frac{51228}{143} = 52773$$

Estimated specificity becomes

$$\text{Spec} = \frac{N_{\text{Script}}}{N_{\text{No sepsis}}} = \frac{52773}{53325} = 0.989$$

PPV is easier to estimate since we only consider patients from group A

$$\text{PPV} = \frac{129}{140} = 0{,}921$$

NPV is somewhat more complicated since we need to include both group B and group C. The ratios from each group can however be combined through the weighted average

$$\text{NPV} = \frac{139}{143} \cdot \frac{1958}{1958 + 51228} + \frac{142}{143} \cdot \frac{51228}{1958 + 51228} = 0.992$$
